# Sleep-administered ketamine/psychedelics: A streamlined strategy to address two challenges in research on ketamine and psychedelics

**DOI:** 10.1192/j.eurpsy.2025.14

**Published:** 2025-02-05

**Authors:** Shokouh Arjmand, Mats B. Lindström, Carl M. Sellgren, Gregers Wegener

**Affiliations:** 1 Department of Physiology and Pharmacology, Karolinska Institutet, Stockholm, Sweden; 2 Translational Neuropsychiatry Unit, Aarhus University, Aarhus, Denmark; 3 Department of Clinical Sciences, Psychiatry, Faculty of Medicine, Lund University, Malmö, Sweden; 4 Centre for Psychiatry Research, Department of Clinical Neuroscience, Stockholm Health Care Services, Karolinska Institutet, and Stockholm Health Care Services, Stockholm, Sweden; 5 Department of Affective Disorders, Aarhus University Hospital - Psychiatry, Aarhus, Denmark

**Keywords:** blinding, ketamine, mechanism of action, psychedelics, rapid-acting antidepressants

## Abstract

The dissociative effects of ketamine and psychedelics might be associated with their rapid antidepressant properties, raising questions about whether these effects are necessary for their therapeutic action. Additionally, the distinct dissociative experiences often reported by patients in clinical trials may reveal whether they receive an active treatment or a placebo, potentially introducing bias into the results. In this viewpoint, we propose administering ketamine/psychedelics to patients during sleep, offering a novel approach to address and explore these challenges.

Although not firmly established, the rapid antidepressant effects of ketamine and psychedelics may be linked to their dissociative/psychoactive properties. Moreover, such effects could inadvertently unblind clinical trials, potentially affecting outcome validity [1–4].

## Masking ketamine and psychedelics treatments allocation by administration during sleep

Successful blinding in double-blind randomized clinical trials (RCTs) is essential for minimizing bias in both patients and investigators. Blinding is especially important in assessing antidepressants, given the use of subjective clinical rating scales and a prominent placebo response. The distinct dissociative and psychoactive effects of ketamine and psychedelics make it questionable if blinding can be maintained. Functional unblinding has also been reported in ketamine trials despite using an active placebo (midazolam), while, to the best of our knowledge, none of the major RCTs on psilocybin report blinding assessments. Given the duration of action, administering ketamine (and psychedelics) while the patient is asleep may, to some extent, overcome this challenge. Theoretically, patients cannot consciously perceive these dissociative effects during sleep, and unblinding can be avoided. This form of “sleep blinding” allows for a more rigorous and unbiased assessment of the antidepressant efficacy.

In a recent trial on the antidepressive effects of ketamine (no superiority over placebo), Lii et al. were also able to effectively mask patient treatment allocation by administrating ketamine under general anesthesia [5]. However, as noted by Lii et al., this masking strategy is impractical and less feasible for most larger placebo-controlled trials. Moreover, there are ongoing controversies regarding the potential antidepressant effects of general anesthetics [6, 7]. From this perspective, sleep-administered ketamine/psychedelics could be an alternative and more practical strategy to blind patients and researchers from treatment allocation.

## Investigating the role of dissociation in the rapid antidepressant effects

Ketamine is renowned for its rapid antidepressant effects, with growing evidence supporting psychedelics, often providing relief within hours or days in contrast to weeks for conventional antidepressants. However, the precise mechanisms underlying this rapid mode of action remain inconclusive. One of the defining features of ketamine and psychedelics treatment, when administered while awake, is their dissociative and psychoactive effects, where patients often feel detached from reality or experience a distortion of perception and might have mystical experiences. While these effects are temporary, they have led researchers to wonder whether this dissociation contributes to psychedelics and ketamine’s rapid antidepressant properties. By administering ketamine (and psychedelics) during sleep and assessing the exerted effects without the influence of dissociation or any mystical experience, the question of whether psychedelics and ketamine’s rapid-acting antidepressant effects require the subjective dissociation/experience induced by the compounds can also be addressed.

Suppose patients show significant improvements in mood without experiencing dissociation/mystical experiences. In that case, this might suggest that dissociation is not integral to psychedelics and ketamine’s mechanism of action in rapidly mitigating symptoms of depression and reducing depression scores obtained by various scales. This would help disentangle the therapeutic effects of the drug from its psychological effects, allowing us to refine our understanding of how ketamine and psychedelics work at a neurobiological level.

This approach could lead to developing treatment options that provide rapid relief from depressive symptomatology without the drawbacks of experiencing dissociation or hallucination and risk of abuse, making the administration and dispensing of such potential rapid-acting antidepressants much easier.

In the case of no response to ketamine and psychedelics treatments during sleep, it is essential to determine whether a possible lack of response stems from the inability of ketamine/psychedelics to evoke dissociative/hallucinogenic experiences or is a result of the improved blinding approach, which may have been compromised in other studies that reported a potentially rapid amelioration of depressive symptoms.

## Research considerations

When designing research clinical trials to this end, we highly encourage EEG monitoring during the infusion/nasal administration of ketamine (or after the administration of psychedelics) to have an understanding of the potential impact of various phases of sleep on the outcome and reduce the confounding factors. When that effect is established, it can be done without EEG monitoring.

Before conducting RCTs involving patients receiving ketamine/psychedelics during sleep, it is advisable to begin with a small population of healthy individuals. Such an approach helps provide valuable information that can be communicated to patients before they participate in trials. It would be beneficial to focus on a population of patients that allows for an easier comparison of the outcomes with those in other published papers. Using the same rating scales can also facilitate more robust comparisons of the outcomes to look both at the impact of dissociative/psychoactive experiences on the therapeutic effect of ketamine and the possible bias arising from improper masking of the active arm.

One critical risk to consider is the potential for nausea and vomiting, which are among the most common side effects of ketamine. Administering ketamine while patients are asleep increases the risk of aspiration due to vomiting. To mitigate this risk, patients should be advised to fast for at least 12 h before receiving ketamine and avoid drinking liquids for 2 h before sleep. Furthermore, considering the onset of the dissociative effects, there might be a need to have a pre-administration of a sedative-hypnotic (e.g., Z-drugs).

## An additional advantage this approach brings

Administering ketamine/psychedelics during sleep could also create a more tolerable treatment model for patients. As a result of psychoactive experiences, some individuals may experience significant anxiety during ketamine infusions or psychedelic sessions [8, 9], which can deter them from seeking treatment despite the potential antidepressant benefits. Administering the drug during sleep may alleviate these anxieties, potentially enhancing patient adherence and overall experience.

This framework highlights not only the innovative approach to improving clinical trials for ketamine/psychedelics but also how it could transform our understanding and future development of rapid-acting antidepressants.

## References

[1] Ballard ED, Zarate CA. The role of dissociation in ketamine’s antidepressant effects. Nat Commun 2020;11:1–7. 33353946 10.1038/s41467-020-20190-4 PMC7755908

[2] Kadriu B, Greenwald M, Henter ID, Gilbert JR, Kraus C, Park LT, et al. Ketamine and serotonergic psychedelics: common mechanisms underlying the effects of rapid-acting antidepressants. Int J Neuropsychopharmacol. 2021;24:8–21. 33252694 10.1093/ijnp/pyaa087 PMC7816692

[3] Hashimoto K. Are “mystical experiences” essential for antidepressant actions of ketamine and the classic psychedelics? Eur Arch Psychiatry Clin Neurosci. 2024;1–14 10.1007/s00406-024-01770-7. PMC12270972 38411629

[4] Muthukumaraswamy SD, Forsyth A, Lumley T. Blinding and expectancy confounds in psychedelic randomized controlled trials. Expert Rev Clin Pharmacol. 2021;14:1133–52. 34038314 10.1080/17512433.2021.1933434

[5] Lii TR, Smith AE, Flohr JR, Okada RL, Nyongesa CA, Cianfichi LJ, et al. Randomized trial of ketamine masked by surgical anesthesia in patients with depression. Nature Mental Health. 2023;1:876–86. 38188539 10.1038/s44220-023-00140-x PMC10769130

[6] Wu G, Xu H. A synopsis of multitarget therapeutic effects of anesthetics on depression. Eur J Pharmacol. 2023;957:176032. 37660970 10.1016/j.ejphar.2023.176032

[7] Tadler SC, Mickey BJ. Emerging evidence for antidepressant actions of anesthetic agents. Curr Opin Anaesthesiol. 2018;31:439–45. 29794854 10.1097/ACO.0000000000000617

[8] Aust S, Gärtner M, Basso L, Otte C, Wingenfeld K, Chae WR, et al. Anxiety during ketamine infusions is associated with negative treatment responses in major depressive disorder. Eur Neuropsychopharmacol. 2019;29:529–38. 30772118 10.1016/j.euroneuro.2019.02.005

[9] Bremler R, Katati N, Shergill P, Erritzoe D, Carhart-Harris RL. Case analysis of long-term negative psychological responses to psychedelics. Sci Rep. 2023;13:1–15. 37749109 10.1038/s41598-023-41145-x PMC10519946

